# Comparative Electrocardiographic Analysis Between Physical Exercise Practitioners and Athletes: A Cross-Sectional Study

**DOI:** 10.3390/ijerph22010078

**Published:** 2025-01-09

**Authors:** Ottavia V. Z. Helbok, Luiz V. A. Sousa, Artur H. Herdy, Gabriel Z. Laporta, Rodrigo D. Raimundo

**Affiliations:** 1 Medical School Research Department, Centro Universitario para o Desenvolvimento do Alto Vale do Itajai (Unidavi), Rio do Sul 89160-932, SC, Brazil; ottavia.helbok@unidavi.edu.br; 2 Study Design and Scientific Writing Laboratory, Centro Universitario FMABC, Santo André 09060-870, SP, Brazil; gabriel.laporta@fmabc.br; 3 Epidemiology and Data Analysis Laboratory, Centro Universitario FMABC, Santo André 09060-870, SP, Brazil; luiz.alcantara@fmabc.net; 4 Institute of Cardiology of Santa Catarina, São José 88103-901, SC, Brazil; arherdy@cardiosport.com.br

**Keywords:** electrocardiogram, physical exercise, athlete’s heart

## Abstract

The trained heart adapts through geometric changes influenced by concentric and eccentric hypertrophy, depending on the predominance of the isometric or dynamic components of the exercise performed. Additionally, alterations in heart rhythm may occur due to increased vagal system activity. Cardiological evaluation with an electrocardiogram (ECG) aims to identify cardiac conditions that could temporarily or permanently disqualify an athlete from competition. This study sought to compare electrocardiographic findings in regular exercisers with those observed in athletes and to correlate these findings with training duration and load. A cross-sectional study was conducted with 154 participants divided into two groups: exercisers (EG) and athletes (AG). Data were collected on exercise type, weekly training time and practice duration. Each participant underwent a resting ECG, analyzed by two independent physicians, with a third review in case of disagreement. The Seattle criteria were applied to categorize ECG changes as physiological, borderline or abnormal. The findings revealed that 75% of athletes exhibited ECG changes, with left and/or right ventricular hypertrophy and incomplete right bundle branch block (IRBBB) being the most prevalent. Age (PR = 0.92; *p* = 0.004) and exercise duration (PR = 1.00; *p* = 0.004) significantly influenced the observed electrocardiographic changes. The majority of both regular exercisers and athletes displayed ECG alterations, with the prevalence increasing with age and training duration.

## 1. Introduction

There are cardiological changes that can occur with the practice of regular physical exercise, and these are not necessarily harmful. In fact, many of these changes are considered physiological for athletes and/or those who practice regular physical exercise. The most common cardiac adaptations are due to increased vagal tone and increased cardiac chambers, primarily through increased muscle mass, influenced by age, gender, ethnicity and sport modality practiced [1,2].

The term “athlete’s heart”, described by Henschen (1899) [3], shows changes in the heart’s geometry, as well as electrical changes. In 1982, the concept of pre-participation assessment emerged in Italy, initially for competitive athletes. After that, there was a drop in the incidence of sudden deaths in competitive individuals in that country. The pre-participation assessment includes an anamnesis, a physical examination and an electrocardiogram to identify heart diseases that could temporarily or even permanently disqualify an athlete.

The prevalence of sudden cardiac death (SCD) in athletes varies, but the most reliable data reveal a prevalence of 1 in 50.000 in young and middle-aged marathoners. In total, 90% of them are male and over 90% of all exercise-related SCDs occur in recreational athletes (exercisers) [2].

After the institution of pre-participation evaluation with ECG, the incidence of sudden death in athletes decreased by 89% [4]. The interpretation of ECGs become more assertive when using specific criteria, published in the *European Heart Journal* in 2018, detailing the international recommendations for electrocardiographic interpretation in athletes and, in this, we find the semaphore system of Seattle criteria, with physiological, borderline, and abnormal or pathological findings [5,6].

In 2016, Araújo and Scharhag [7] published an editorial in the *Scandinavian Journal of Medicine and Sports Sciences* on the definition of “athlete”, noting that this term was introduced about 40 years ago, but a clear definition is still lacking. In essence, athletes are considered to be very well conditioned in relation to the general population in the same sex and age group. However, in general, athletes tend to represent a non-homogeneous group in several aspects, including morphological, physiological, psychological and clinical aspects. The authors proposed at the time that in order to use the term athlete, the individual should meet the following four criteria: (1) be in training with the intention of improving performance or results; (2) actively participate in sports competitions; (3) be formally registered with a sports federation as a competitor; and (4) have training and competitions as their main activity, spending several hours of their day on this. Adopting this definition, expressions such as recreational or leisure athlete will be dispensed with, and the term “exercisers” will be used.

Considering the current scenario of physical exercise practitioners (exercisers), it is hypothesized that there are electrocardiographic changes in these individuals that resemble athletes’ electrocardiograms. This study seeks to identify which changes may occur in the electrocardiograms of individuals who practice regular physical exercise. It is widely believed that the electrocardiographic alterations of athletes are not limited only to this group, but also apply to exercisers, thus providing better guidance for those who present alterations that would be considered pathological.

Raising awareness among exercisers of the real need for an adequate medical evaluation, with regular follow-up, improves quality of life, minimizing health risks [8]. In addition, by knowing what changes can occur in the electrocardiograms of exercisers, we can optimize medical care, thus adding value to sports and exercise medicine.

The research aims to compare electrocardiographic changes in exercisers with the ones found in athletes, correlate these changes with time and training load, and in this way identify whether the non-athlete population will suffer the same alterations as athletes.

## 2. Materials and Methods

### 2.1. Study Design

This was a cross-sectional study carried out in the Sports Medicine Department of the Centro Universitario para o Desenvolvimento do Alto Vale do Itajaí (Santa Catarina/Brazil) from June 2022 to April 2023.

### 2.2. Participants

The individuals included in the study were categorized into two groups: the exerciser group (EG) and the athlete group (AG). The eligibility criteria for the EG were as follows: (1) involvement in physical activities for a period of more than 12 consecutive months; (2) performing at least 150 min of physical exercise per week, as recommended by the World Health Organization (WHO); (3) aged 18 years or older. To be included in the AG, the criteria were as follows: (1) involvement in physical activities for more than 12 months; (2) being officially registered with a sports federation; (3) active participation in competitions in their sport and having that sport as their main occupation; (4) aged 18 years or older.

### 2.3. Assessment Variables and Methods

**Figure 1:**
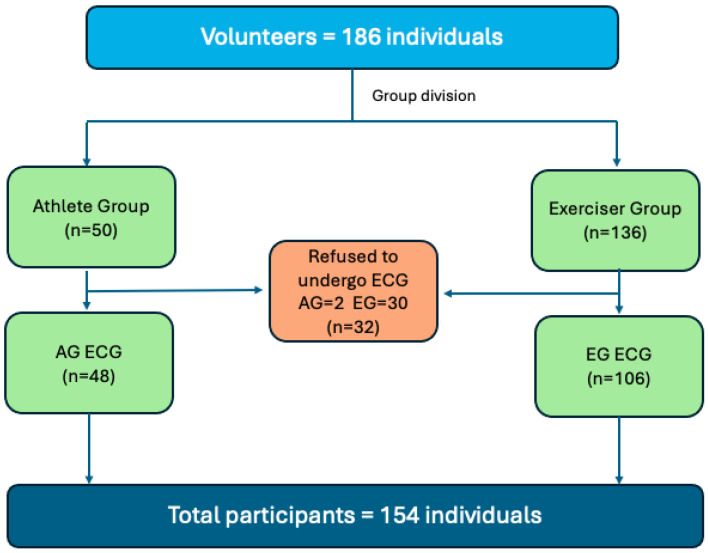
Inclusion flowchart for participants aged 18 years or older. Athletes were defined as (1) involved in physical activities for a period of more than 12 months; (2) officially registered with a sports federation; (3) active participants in competitions in their sport; and (4) having that sport as their main occupation. Exercisers did not meet these criteria. Participants who did not respond or refused to undergo the ECG were withdrawn from the study.

Study participants were contacted following approval by the Ethics and research Committee (approval number: 5,493,097). After signing an Informed Consent Form (ICF), the initial evaluation form was completed. This form included three sections: the first section contained the ICF, the second section comprised 16 questions for identification and sociodemographic characteristics, and the third section included an international physical activity questionnaire (IPAQ-short form) [9], adapted for physical exercises, with 11 questions. The questionnaire aimed to categorize the variables days/week, hours/week and minutes/week, as well as the intensity of the exercise performed. Initially, this semi-structured questionnaire was filled out, identifying individuals who practiced physical exercise for the weekly duration recommended in the inclusion criteria and for the division of the groups (Figure 1).

### 2.4. Electrocardiograms

**Figure 2:**
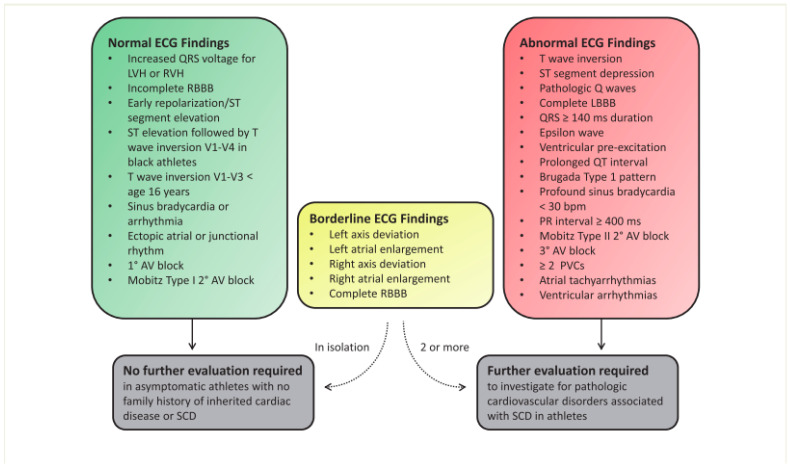
International consensus standards for electrocardiogram interpretation in athletes (from Sharma et al. European heart Journal (2018) 39. 1466–1480 (6)). AV, atrioventricular block; LBBB, left bundle branch block; LHV, left ventricular hypertrophy; RBBB, right bundle branch block; RVH, right ventricular hypertrophy; PVC, premature ventricular contraction; SCD, sudden cardiac death.

After data collection and the categorization of each individual, participants underwent an electrocardiogram at rest, and these were reported and evaluated by two independent physicians. In case of disagreement, results were analyzed by a third professional for consensus by at least two evaluators. The findings of the ECGs were categorized according to the Seattle criteria (physiological, borderline or abnormal), also described in the international recommendations for evaluating the electrocardiograms of athletes [5,6]. (Figure 2).

### 2.5. Potential Sources of Bias

As it is a self-completed form, the information on the amount and duration of physical exercise practiced per week may not be reliable. To reduce this risk, when performing the resting electrocardiogram, the data on the form were confirmed with the participant, and information such as weight and height were reassessed for accuracy.

### 2.6. Sample Size

Based on prevalence studies by Okoh et al. (2024), Koufou et al. (2022) and Stazi (2019) [10,11,12], the sample size for the study was calculated using the following assumptions. The required sample size was estimated to be 120 patients, based on our city population, using an estimated 60% prevalence of electrocardiographic changes in athletes, 95% as the confidence level, 5% for precision and 5% for the dropout rate.

### 2.7. Statistical Analysis

Qualitative variables were expressed as absolute frequencies and percentages, while quantitative variables were described as mean and standard deviation. The normality of continuous variables was verified using the Shapiro–Wilk test.

Pearson’s chi-square test was applied to analyze the associations between the following:

(a) Athlete group (GA) and exerciser group (EG) with ECG abnormalities, revealing a significantly higher prevalence of abnormalities in athletes (*p* < 0.001).
(b) The electrocardiogram alteration type (such as ventricular hypertrophy, incomplete right bundle branch block, or sinus bradycardia) and the status of the athlete or exerciser, according to the Seattle criteria.
(c) The weekly exercise duration (up to 150 min, 150–300 min and more than 300 min) and the presence of ECG abnormalities in both groups, with statistically significant results (*p* < 0.01).
(d) The intergroup comparison of exercise time and the prevalence of ECG abnormalities in the AG and EG (*p* < 0.01).

Poisson regression was applied to assess the relationship between several predictor variables and ECG changes. The variables included in the model were as follows:

(a) Age: age was significantly associated with ECG changes, with an increase in prevalence as age advanced (*p* = 0.004).
(b) Weekly exercise duration: the relationship between exercise time (in minutes per week) and changes in the ECG was evaluated, adjusting for age and total practice time. Individuals who practiced more than 300 min of weekly exercise had a higher prevalence of alterations (*p* < 0.001).
(c) Total exercise duration: the total exercise time (in months) was also included as a predictor variable, and a significant association was observed with ECG changes (*p* = 0.009). This regression allowed for the calculation of the prevalence ratio (PR) of electrocardiographic alterations, adjusting for the variables age and duration of exercise, providing a clearer view of the impact of these variables on ECG alterations.

All analyses were adjusted for possible confounders, such as gender and type of exercise. The level of significance was set at 5% (*p* < 0.05), and all analyses were performed using the Stata software, version 18.0^®^.

## 3. Results

Of the 154 individuals included in the study, 48 (31.16%) were athletes (AG) and 106 (68.83%) were exercisers (EG). The sample showed an average age of 33.55 (SD = 10.67) years. Only five individuals, all from the EG, reported using anabolic steroids. While anabolic steroid use poses significant risks to the cardiovascular system, none of these individuals exhibited electrocardiographic alterations. Nevertheless, they were provided with guidance on the associated risks. The complete characterization of the sample is found in Table 1.

The time spent exercising per week and type of sport practiced were evaluated. The analysis of the time participants spent exercising per week showed an average of more than 300 min/week. The most practiced sport in the AG was soccer. In the EG, CrossFit prevailed (17.92%), followed by Volleyball (16.04%) and Jiu-jitsu (11.32%). All modalities are shown in Table 2.

When evaluating the ECG, first it was verified whether the ECG had any changes. In the AG, 75% of individuals had some changes, while in the EG 61.32% had no changes (*p* < 0.001). The changes found were categorized into ECGs with physiological changes, ECGs with borderline changes and ECGs with pathological changes, according to the Seattle criteria published in 2017 (5). Within the ECG findings with physiological changes, the presence of LVH prevailed (41.56%), followed by incomplete right bundle branch block (IRBBB) (23.38%) and sinus bradycardia (15.58%) (*p* < 0.001). Evaluating the separate groups, in the AG, the finding of LVH prevailed (69.44%), followed by sinus bradycardia (13.89%). In the EG, IRBBB prevailed (36.59%), followed by LVH (17.07%) and sinus bradycardia (17.07%). Table 3a,b summarizes these findings.

All study participants who had alterations considered pathological by the Seattle criteria, or who had two borderline alterations, were referred for a more detailed cardiological evaluation. At the time of writing, we have not received definitive feedback; we only know that the individual who had an ECG with pre-excitation was instructed not to participate in competitions until the end of the pertinent cardiological evaluations.

Table 4 shows the duration of physical exercise performed in min/week and the findings of changes on the ECG. The results that showed statistical significance were in the GA, in participants who practiced between 150 and 300 min/week of physical exercise; none of them presented an abnormal ECG (*p* = 0.002).

Table 5 shows the intergroup analysis of individuals who presented changes in the ECG and the duration of physical exercise practiced per week. It was observed that there was statistical significance in individuals who practiced between 150 and 300 min/week of physical exercise (*p* = 0.001) and more than 300 min/week of physical exercise (*p* = 0.002).

Multivariate logistic regression (Table 6) showed that both age and exercise duration directly interfere with the electrocardiographic changes found. The older the individual, and the longer the exercise is practiced, the greater the prevalence of electrocardiographic changes. Nonetheless, the model proved to be fragile with low explanatory power.

## 4. Discussion

This study showed that 75% of individuals in the athlete group (AG) showed some change in their electrocardiogram (ECG), while in the exerciser group (EG) the percentage was 38.68%. Among the changes observed in the AG, left and/or right ventricular hypertrophy and incomplete right bundle branch block (IRBBB) were predominant. In the EG, the most common change was the IRBBB, followed by left and/or right ventricular hypertrophy and sinus bradycardia. It was observed that age and the duration of physical exercise directly influence electrocardiographic changes, with a greater prevalence of changes as age and duration of practice increase.

International recommendations for ECG interpretation in athletes, such as traffic light-style criteria, facilitate the categorization of changes found and emphasize the need for further investigation. Previous studies corroborate our research findings, such as the studies by Drezner et al. (2017) [5] and Sharma et al. (2018) [6], who reported left ventricular hypertrophy (LVH) as a common change among athletes. This was a predominant finding within this study. Recent reviews, such as the one prepared by Ujeyl and Niederseer (2023) [13], indicate that 45% of healthy athletes present LVH, with only 1.8% requiring further investigation, a higher prevalence than the one observed in this study. Moreover, these reviews highlight the importance of the presence of multiple premature ventricular contraction (PVC) on the baseline ECG, a finding not observed in participants of this research.

Claessen et al. (2020) [14] studied early repolarization in young athletes, finding a prevalence of 24% among athletes, a significantly higher rate than that found in this study (2.78% in the AG and 7.32% in the EG). Jakubiak et al. (2017) [15] evaluated endurance athletes and identified age, training duration and male gender as predictors of changes in the ECG. These findings are in line with the study results, which show age and training duration as factors that increase the prevalence of electrocardiographic changes. Panhuyzen-Goedkoop et al. (2020) [16] reported that 5.4% of master athletes had pathological changes on ECGs, with a prevalence of 2.2% of T wave inversion, compared to the 5.19% found in this cross-sectional study.

Hedman et al. (2020) [17] studied the correlation between left ventricular (LV) mass and the ECG voltage, concluding that the distance from the chest wall to the LV is a weak determinant of ECG voltage and pathological hypertrophy. Liu et al. (2023) [18] evaluated female athletes and observed a longer QRS complex duration and right axis deviation in endurance athletes. In the current study, 8.33% of the AG and 7.32% of the EG presented right axis deviation without statistical significance.

Liu et al. (2023) [18] reported 6.1% and 5.9% prevalence of LVH in endurance and strength athletes, respectively, which contrasts with the higher rates found in this study—69.4% in the AG and 17.07% in the EG. This discrepancy may stem from women’s lower susceptibility to electrocardiographic changes, as highlighted by D’Ascenzi et al. (2020) [19] and Wooten et al. (2021) [20], and the fact that only 24.68% of participants in this study were women. D’Ascenzi et al. (2020) [19] further explored sex-based ECG differences, reporting LVH in 32.9% of male and 9% of female athletes, underscoring the influence of sex. Although the present study identified an overall LVH prevalence of 41.5%, it did not aim to assess gender differences specifically. Additionally, Ferrari et al. (2024) [21] observed a 3% prevalence of abnormal ECGs in soccer athletes, predominantly characterized by T wave inversion, aligning with this study’s findings of 5.56% abnormal ECGs in the AG.

This study had some limitations. First, the sample of the two groups was not equivalent in absolute numbers and sport modalities, preventing an assessment of differences among modalities. Second, data collection using a self-completed form may have introduced inaccuracies, mitigated by reassessment at the time of the ECG.

Finally, the knowledge of possible electrocardiographic changes in athletes and those who practice regular exercises is crucial. Furthermore, a periodic evaluation of these individuals is vital for the early identification of changes, which are not limited to athletes, but can also occur in those who practice regular physical exercise. And as we mention in Introduction, the electrocardiographic alterations of athletes are not limited to only this group, but also apply to exercisers, as it was observed that age and duration of the practice directly influence these alterations in both groups. And knowing this, we can provide better guidance for those who practice exercises and present any alterations that would be considered pathological or not.

## 5. Conclusions

Most athletes and more than a third of those who exercise regularly present electrocardiographic changes related to physical activity. In athletes, the most common are left and/or right ventricular hypertrophy, while in regular exercisers, incomplete right bundle branch block stands out. It was observed that these changes are more prevalent in older individuals and those who have practiced exercise for a longer period, highlighting the influence of these factors on the manifestation of these conditions. This study highlights the importance of conducting regular evaluations, including anamnesis, physical examinations and resting ECGs, for both regular exercisers and athletes to provide better guidance and help prevent events such as SCD.

## Figures and Tables

**Table 1:** Sample characterization (N = 154).

Variables	AG Average (sd)	EG Average (sd)	Total Average (sd)	*p*-Value
	n = 48	n = 106	N = 154	
Age	27.14 (9.48)	36.46 (9.93)	33.55 (10.67)	<0.001 *
Weight (kg)	77.34 (16.53)	79.18 (16.33)	78.61 (16.36)	0.519
Height (m)	1.78 (0.09)	1.73 (0.10)	1.75 (0.10)	0.015 *
BMI	24.18 (3.51)	26.09 (4.18)	25.50 (4.09)	0.006 *
Moderate exercise in minutes	337.81 (196.97)	250.66 (148.09)	277.82 (169.14)	0.002 *
Vigorous exercise in minutes	301.25 (152.83)	168.72 (110.98)	210.03 (139.36)	<0.001 *
Total exercise time in months	173.25 (112.04)	145.80 (121.87)	154.35 (119.21)	0.186
		n (%)		*p*-value
Male	44 (91.67)	72 (67.92)	116 (75.32)	0.002 *
Up to 150 min/week	2 (4.17)	7 (6.60)	9 (5.84)	0.550
150 to 300 min/week	3 (6.23)	40 (37.74)	43 (27.92)	<0.001 *
More than 300 min/week	43 (89.58)	59 (55.66)	102 (66.23)	<0.001 *
Race				<0.001 *
White	26 (54.17)	92 (86.79)	117 (76.47)	
Black	16 (33.33)	4 (3.81)	20 (13.07)	
Mulatto	5 (10.42)	9 (8.57)	14 (9.15)	
Indigenous	1 (2.08)	0 (0.00)	1 (0.65)	
Eastern	0 (0.00)	1 (0.95)	1 (0.65)	

Notes: AG: athlete group; EG: exerciser group; min/week: minutes per week; sd: standard deviation; n: relative sample number; N: absolute sample number; %: frequency. Statistical method employed: * Pearson chi-square. The * *p*-value ≤ 0.05 was considered statistically significant.

**Table 2:** Exercise duration modalities.

Exercise Time and Modalities	AG n (%)	EG n (%)	Total n (%)	*p*-Value *
	n = 48	n = 106	N = 154	
Up to 150 min/week	2 (4.17)	7 (6.60)	9 (5.84)	0.550
150 to 300 min/week	3 (6.23)	40 (37.74)	43 (27.92)	<0.001 *
More than 300 min/week	43 (89.58)	59 (55.66)	102 (66.23)	<0.001 *
Sports modalities				
Soccer	28 (58.33)	1 (0.94)	29 (18.83)	
CrossFit	1 (2.08)	19 (17.92)	20 (12.99)	
Volleyball	1 (2.08)	17 (16.04)	18 (11.69)	
Jiu-jitsu	0 (0.00)	12 (11.32)	12 (7.79)	
Muay Thai	2 (4.17)	9 (8.49)	11 (7.14)	
Bodybuilding	0 (0.00)	9 (8.49)	9 (5.84)	
Futsal	3 (6.25)	6 (5.66)	9 (5.84)	
Basketball	2 (4.17)	6 (5.66)	8 (5.19)	
Running	1 (2.08)	6 (5.66)	7 (4.55)	<0.001 *
Judo	3 (6.25)	3 (2.83)	6 (3.90)	
Triathlon	0 (0.00)	5 (4.72)	5 (3.25)	
Cycling	2 (4.16)	3 (2.83)	5 (3.24)	
Swimming	0 (0.00)	4 (3.77)	4 (2.60)	
Handball	1 (2.08)	3 (2.83)	4 (2.60)	
Athletics	3 (6.25)	0 (0.00)	3 (1.95)	
Functional	0 (0.00)	2 (1.89)	2 (1.30)	
Karate	0 (0.00)	1 (0.94)	1 (0.65)	
Beach Tennis	1 (2.08)	0 (0.00)	1 (0.65)	

Notes: AG: athlete group; EG: exerciser group; min/week: minutes per week. n: relative sample number; N: absolute sample number; %: frequency; Statistical Method Used: Pearson chi-square. The * *p*-value ≤ 0.05 was considered statistically significant.

**Table 3:** (a) Analysis of the presence of ECG alterations; (b) ECG alterations analysis.

**(a)**				
**ECG Analysis**	**AG n (%)**	**EG n (%)**	**Total n (%)**	***p*-Value** *
	**n = 48**	**n = 106**	**N = 154**	
**ECG**				
With no changes	12 (25.0)	65 (61.32)	77 (50.0)	<0.001 *
Altered ECG	36 (75.0)	41 (38.68)	77 (50.0)	
**(b)**				
**ECG Alterations Analysis**	**AG n (%)**	**EG n (%)**	**Total n (%)**	***p*-Value**
	**n = 36**	**n = 41**	**N = 77**	
Physiological				<0.001 *
LVH and/or RVH	25 (69.44)	7 (17.07)	32 (41.56)	
IRBBB	3 (8.33)	15 (36.59)	18 (23.38)	
Early repolarization	1 (2.78)	3 (7.32)	4 (5.19)	
T wave inversion V1-V4	1 (2.78)	1 (2.44)	2 (2.60)	
Bradicardia sinusal	5 (13.89)	7 (17.07)	12 (15.58)	
1° AV block	0 (0.00)	2 (4.88)	2 (2.60)	
Borderline				0.477
Left axis deviation	0 (0.00)	2 (4.88)	2 (2.60)	
Left atrial enlargement	1 (2.78)	5 (12.20)	6 (7.79)	
Right axis deviation	3 (8.33)	3 (7.32)	6 (7,79)	
Abnormal or pathological				0.571
T wave inversion	2 (5.56)	2 (4.88)	4 (5,19)	
Ventricular pre-excitation	0 (0.00)	1 (2.44)	1 (1,30)	

Note: AG: athlete group; EG: exerciser group; ECG: electrocardiogram; n: relative sample number; N: absolute sample number; %: frequency; LVH: left ventricle hypertrophy; RVH: right ventricle hypertrophy; IRBBB: incomplete right bundle branch block; AV: atrioventricular. Statistical method employed: Pearson chi-square. The * *p*-value ≤ 0.05 was considered statistically significant.

**Table 4:** Analysis of exercise time per week.

Exercise Time/Week	No Changes In ECG	Altered ECG	Total	*p*-Value
	n (%)	n (%)	n (%)	
Exercisers (EG)				
Up to 150 min/week	4 (57.14)	3 (42.86)	7 (100.0)	0.814
150–300 min/week	29 (72.50)	11 (27.50)	40 (100.0)	0.066
More than 300 min/week	32 (54.24)	27 (45.76)	59 (100.0)	0.093
Athletes (AG)				
Up to 150 min/week	0 (0.0)	2 (100.0)	2 (100.0)	0.404
150–300 min/week	0 (0.0)	2 (100.0)	2 (100.0)	0.002 *
More than 300 min/week	3 (100.0)	0 (00.0)	3 (100.0)	0.056

Notes: ECG: electrocardiogram; min/week: minutes per week; n: relative sample number; %: frequency. Statistical method employed: Pearson chi-square. The * *p*-value ≤ 0.05 was considered statistically significant.

**Table 5:** Intergroup analysis of individuals with ECG changes.

Exercise Time/Week	EG n (%)	AG n (%)	Total n (%)	*p*-Value
Up to 150 min/week	3 (60.00)	2 (40.00)	5 (100.00)	0.754
150–300 min/week	11 (100.0)	0 (00.00)	11 (100.00)	0.001 *
More than 300 min/week	27 (44.26)	34 (55.74)	61 (100.00)	0.002 *

Notes: ECG: electrocardiogram; min/week: minutes per week; n: relative sample number; %: frequency. Statistical method employed: Pearson chi-square. The * *p*-value ≤ 0.05 was considered statistically significant.

**Table 6:** Poisson regression.

Variable	PR	*p*-Value	IC95%
ECG change	1.99	<0.001	1.44 to 2.59
150–300 min/week	0.64	<0.001	0.59 to 0.69
Above 300 min	1.38	<0.001	1.17 to 1.63
Total exercise time	1.00	0.70	0.99 to 1.00
General LVH or RVH	1.55	0.001	1.22 to 1.98
Age	0.92	0.004	0.87 to 0.97
Exercise time in months	1.00	0.009	1.00 to 1.01
General IRBBB	1.02	0.88	0.77 to 1.34
Age	1.09	0.001	1.03 to 1.16
Exercise time in months	0.99	0.004	0.98 to 0.99

Notes: ECG: electrocardiogram; IRBBB: incomplete right bundle branch block; LVH: left ventricular hypertrophy; min/week: minutes per week; RVH: right ventricular hypertrophy; PR: prevalence ratio.

## Data Availability

Data from this research will be shared upon request to the corresponding author (rodrigo.raimundo@fmabc.br).
